# A Protocol for the Use of Case Reports/Studies and Case Series in Systematic Reviews for Clinical Toxicology

**DOI:** 10.3389/fmed.2021.708380

**Published:** 2021-09-06

**Authors:** Aboubakari Nambiema, Grace Sembajwe, Juleen Lam, Tracey Woodruff, Daniele Mandrioli, Nicholas Chartres, Marc Fadel, Adrien Le Guillou, Remi Valter, Marie Deguigne, Marion Legeay, Chloe Bruneau, Gaël Le Roux, Alexis Descatha

**Affiliations:** ^1^Univ Angers, CHU Angers, Univ Rennes, INSERM, EHESP, Institut de Recherche en Santé, Environnement et Travail-UMR_S 1085, Angers, France; ^2^Department of Occupational Medicine, Epidemiology and Prevention, Donald and Barbara Zucker School of Medicine, Northwell Health, Feinstein Institutes for Medical Research, Hofstra University, Great Neck, NY, United States; ^3^Department of Health Sciences, University of California, San Francisco and California State University, Hayward, CA, United States; ^4^Program on Reproductive Health and the Environment, University of California, San Francisco, San Francisco, CA, United States; ^5^Cesare Maltoni Cancer Research Center, Ramazzini Institute, Bologna, Italy; ^6^Department of Research and Public Health, Reims Teaching Hospitals, Robert Debré Hospital, Reims, France; ^7^CHU Angers, Univ Angers, Poisoning Control Center, Clinical Data Center, Angers, France

**Keywords:** toxicology, epidemiology, public health, protocol, systematic review, case reports/studies, case series

## Abstract

**Introduction:** Systematic reviews are routinely used to synthesize current science and evaluate the evidential strength and quality of resulting recommendations. For specific events, such as rare acute poisonings or preliminary reports of new drugs, we posit that case reports/studies and case series (human subjects research with no control group) may provide important evidence for systematic reviews. Our aim, therefore, is to present a protocol that uses rigorous selection criteria, to distinguish high quality case reports/studies and case series for inclusion in systematic reviews.

**Methods:** This protocol will adapt the existing Navigation Guide methodology for specific inclusion of case studies. The usual procedure for systematic reviews will be followed. Case reports/studies and case series will be specified in the search strategy and included in separate sections. Data from these sources will be extracted and where possible, quantitatively synthesized. Criteria for integrating cases reports/studies and case series into the overall body of evidence are that these studies will need to be well-documented, scientifically rigorous, and follow ethical practices. The instructions and standards for evaluating risk of bias will be based on the Navigation Guide. The risk of bias, quality of evidence and the strength of recommendations will be assessed by two independent review teams that are blinded to each other.

**Conclusion:** This is a protocol specified for systematic reviews that use case reports/studies and case series to evaluate the quality of evidence and strength of recommendations in disciplines like clinical toxicology, where case reports/studies are the norm.

## Introduction

Systematic reviews are routinely relied upon to qualitatively synthesize current knowledge in a subject area. These reviews are often paired with a meta-analysis for quantitative syntheses. These qualitative and quantitative summaries of pooled data, collectively evaluate the quality of the evidence and the strength of the resulting research recommendations.

There currently exist several guidance documents to instruct on the rigors of systematic review methodology: (i) the Cochrane Collaboration, Preferred Reporting Items for Systematic Reviews and Meta-Analyses (PRISMA) statement and PRISMA-P (for protocols) that offer directives on data synthesis; and (ii) the Grading of Recommendations, Assessment, Development and Evaluations (GRADE) guidelines that establish rules for the development of scientific recommendations (1–5). This systematic review guidance is based predominantly on clinical studies, where randomized controlled trials (RCTs) are the gold standard. For that reason, a separate group of researchers has designed the Navigation Guide, specific to environmental health studies that are often observational (6, 7). To date, systematic review guidelines (GRADE, PRISMA, PRISMA-P, and Navigation Guide) remove case reports/studies and case series (human subjects research with no control group) from consideration in systematic reviews, in part due to the challenges in evaluating the internal validity of these kinds of study designs. We hypothesize, however, that under certain circumstances, such as in rare acute poisonings, or preliminary reports of new drugs, some case reports and case series may contribute relevant knowledge that would be informative to systematic review recommendations. This is particularly important in clinical settings, where such evidence could potentially change our understanding of the screening, presentation, and potential treatment of rare conditions, such as poisoning from obscure toxins. The Cochrane Collaboration handbook states that “*for some rare or delayed adverse outcomes only case series or case-control studies may be available. Non-randomized studies of interventions with some study design features that are more susceptible to bias may be acceptable for evaluation of serious adverse events in the absence of better evidence, but the risk of bias must still be assessed and reported*” (8). In addition, the Cochrane Adverse Effects group has shown that case studies may be the best settings in which to observe adverse effects, especially when they are rare and acute (9). We believe that there may be an effective way to consider case reports/studies and case series in systematic reviews, specifically by developing specific criteria for their inclusion and accounting for their inherent bias.

We propose here a systematic review protocol that has been specifically developed to consider the inclusion and integration of case reports/studies and case series. Our main objective is to create a protocol that is an adaptation of the Navigation Guide (6, 10) that presents methodology to examine high quality case reports/studies and case series through cogent inclusion and exclusion criteria. This methodology is in concordance with the Cochrane Methods for Adverse Effects for scoping reviews (11).

## Methods

This protocol was prepared in accordance with the usual structured methodology for systematic reviews (PRISMA, PRISMA-P, and Navigation guide) (3–7, 10). The protocol will be registered on an appropriate website, such as one of the following:

(i) The International Prospective Register of Systematic Reviews (PROSPERO) database (https://www.crd.york.ac.uk/PROSPERO/) is an international database of prospectively registered systematic reviews in health and social welfare, public health, education, crime, justice, and international development, where there is a health-related outcome. It aims to provide a comprehensive listing of systematic reviews registered at inception to help avoid duplication and reduce opportunity for reporting bias by enabling comparison of the completed review with what was planned in the protocol. PROSPERO accepts registrations for systematic reviews, rapid reviews, and umbrella reviews. Key elements of the review protocol are permanently recorded and stored.
(ii) The Open Science Framework (OSF) platform (https://osf.io/) is a free, open, and integrated platform that facilitates open collaboration in research science. It allows for the management and sharing of research project at all stages of research for broad dissemination. It also enables capture of different aspects and products of the research lifecycle, from the development of a research idea, through the design of a study, the storage and analysis of collected data, to the writing and publication of reports or research articles.
(iii) The Research Registry (RR) database (https://www.researchregistry.com/) is a one-stop repository for the registration of all types of research studies, from “first-in-man” case reports/studies to observational/interventional studies to systematic reviews and meta-analyses. The goal is to ensure that every study involving human participants is registered in accordance with the 2013 Declaration of Helsinki. The RR enables prospective or retrospective registrations of studies, including those types of studies that cannot be registered in existing registries. It specifically publishes systematic reviews and meta-analyses and does not register case reports/studies that are not first-in-man or animal studies.

Any significant future changes to the protocol resulting from knowledge gained during the development stages of this project will be documented in detail and a rationale for all changes will be proposed and reported in PROSPERO, OSF, or RR.

The overall protocol will differentiate itself from other known methodologies, by defining two independent teams of reviewers: a classical team and a case team. The classical team will review studies with control groups and an acceptable comparison group (case reports/studies and case series will be excluded). In effect, this team will conduct a more traditional systematic review where evidence from case reports/studies and case series are not considered. The case team will review classical studies, case reports, and case series. This case team will act as a comparison group to identify differences in systematic review conclusions due to the inclusion of evidence from case reports/studies and case series. Both teams will identify studies that meet specified inclusion criteria, conduct separate analyses and risk of bias evaluations, along with overall quality assessments, and syntheses of strengths of evidence. Each team will be blinded to the results of the other team throughout the process. Upon completion of the systematic review, results from each team will be presented, evaluated, and compared.

### Patient and Public Involvement

No patient involved.

### Eligibility Criteria

Studies will be selected according to the criteria outlined below.

#### Study Designs

Studies of any design reported in any translatable language to English by online programs (e.g., Google Translate) will be included at the beginning. These studies will span interventional studies with control groups (Randomized Controlled Trials: RCTs), as well as observational studies with and without exposed groups. All observational studies will be eligible for inclusion in accordance with the objectives of this systematic review. Thereafter, only the case team will include cases reports/studies and case series, as specified in their search strategy. The case team will include a separate section for human subjects research that has been conducted with no control groups.

#### Type of Population

All types of studies examining the general adult human population or healthy adult humans will be included. Studies that involve both adults and children will also be included if data for adults are reported separately. Animal studies will be excluded for the methodological purpose of this (case reports/studies and case series) protocol given that the framework for systematic reviews in toxicology already adequately retrieves this type of toxin data on animals.

#### Inclusion/Exclusion Criteria

Studies of any design will be included if they fulfill all the eligibility criteria. To be integrated into the overall body of evidence, cases reports/studies and case series must meet pre-defined criteria indicating that they are well-documented, scientifically rigorous, and follow ethical practices, under the CARE guidelines (for **Ca**se **Re**ports) (12, 13) and the Joanna Briggs Institute (JBI) Critical Appraisal Checklist for Case reports/studies and for Case Series (14, 15) that classify case reports/studies in terms of completeness, transparency and data analysis. Studies that were conducted using unethical practices will be excluded.

#### Type of Exposure/Intervention

Either the prescribed treatment or described exposure to a chemical substance (toxin/toxicant) will be detailed here.

#### Type of Comparators

In this protocol we plan to compare two review methodologies: one will include and the other will exclude high quality case reports/studies and case series; these two review methodologies will be compared. The comparator will be (the presence or absence of) an available control group that has been specified and is acceptable scientifically and ethically.

#### Type of Outcomes

The outcome of mortality or morbidity related to the toxicological exposure, will be detailed here.

### Information Sources and Search Strategy

There will be no design, date or language limitations applied to the search strategy. A systematic search in electronic academic databases, electronic grey literature, organizational websites, and internet search engines will be performed. We will search at least the following major databases:

- ***Electronic academic databases***: Pubmed, Web of Sciences, Toxline, Poisondex, and databases specific to case reports/studies and case series (e.g., PMC, Scopus, Medline) (13)
- ***Electronic grey literature databases***: OpenGrey (http://www.opengrey.eu/), grey literature Report (http://greylit.org/)
- ***Organizational websites***: AHRQ Patient Safety Network (https://psnet.ahrq.gov/webmm), World Health Organization (www.who.int)
- ***Internet search engines***: Google (https://www.google.com/), GoogleScholar (https://scholar.google.com/).

### Study Records

Following a systematic search in all the databases above, each of the two independent teams of reviewers (the classical team and the case team) will, respectively, upload separately and in accordance with the eligibility criteria, the literature search results to the systematic review management software, “Covidence,” a primary screening and data extraction tool (16).

All study records identified during the search will be downloaded and duplicate records will be identified and deleted. Thereafter, two research team members will independently screen the titles and abstracts (step 1) and then the full texts (step 2) of potentially relevant studies for inclusion. If necessary, information will be requested from the publication authors to resolve questions about eligibility. Finally, any disagreements that may potentially exist between the two research team members will be resolved first by discussion and then by consulting a third research team member for arbitration.

If a study record identified during the search was authored by a reviewing research team member, or that team member participated in the identified study, that study record will be re-assigned to another reviewing team member.

### Data Collection Process, Items Included, and Prioritization if Needed

All reviewing team members will use standardized forms or software (e.g., Covidence), and each review member will independently extract the data from included studies. If possible, the extracted data will be synthesized numerically. To ensure consistency across reviewers, calibration exercises (reviewer training) will be conducted prior to starting the reviews. Extracted information will include the minimum study characteristics (study authors, study year, study country, participants, intervention/exposure, outcome), study design (summary of study design, comparator, models used, and effect estimate measure) and study context (e.g., data on simultaneous exposure to other risk factors that would be relevant contributors to morbidity or mortality). As specified in the section on study records, a third review team member will resolve any conflicts that arise during data extraction that are not resolved by consensus between the two initial data extractors.

Data on potential conflict of interest for included studies, as well as financial disclosures and funding sources, will also be extracted. If no financial statement or conflict of interest declaration is available, the names of the authors will be searched in other studies published within the previous 36 months and in other publicly available declarations of interests, for funding information (17, 18).

### Risk of Bias Assessment

To assess the risk of bias within included studies, the internal validity of potential studies will be assessed by using the Navigation Guide tool (6, 19), which covers nine domains of bias for human studies: (a) source population representation; (b) blinding; (c) exposure or intervention assessment; (d) outcome assessment; (e) confounding; (f) incomplete outcome data; (g) selective outcome reporting; (h) conflict of interest; and (i) other sources of bias. For each section of the tool, the procedures undertaken for each study will be described and the risk of bias will be rated as “*low risk”; “probably low risk”; “probably risk”; “high risk”; or “not applicable.”* Risk of bias on the levels of the individual study and the entire body of evidence will be assessed. Most of the text from these instructions and criteria for judging risk of bias has been adopted verbatim or adapted from one of the latest Navigation Guide systematic reviews used by WHO/ILO (6, 19, 20).

For case reports/studies and case series, the text from these instructions and criteria for judging risk of bias has been adopted verbatim or adapted from one of the latest Navigation Guide systematic reviews (21), and is given in Supplementary Material. Specific criteria are listed below. To ensure consistency across reviewers, calibration exercises (reviewer training) will be conducted prior to starting the risk of bias assessments for case reports/studies and case series.

#### Are the Study Groups at Risk of Not Representing Their Source Populations in a Manner That Might Introduce Selection Bias?

The source population is viewed as the population for which study investigators are targeting their study question of interest.

Examples of considerations for this risk of bias domain include: (1) the context of the case report; (2) level of detail reported for participant inclusion/exclusion (including details from previously published papers referenced in the article), with inclusion of all relevant consecutive patients in the considered period; (14, 15) (3) exclusion rates, attrition rates and reasons.

#### Were Exposure/Intervention (Toxic, Treatment) Assessment Methods Lacking Accuracy?

The following list of considerations represents a collection of factors proposed by experts in various fields that may potentially influence the internal validity of the exposure assessment in a systematic manner (not those that may randomly affect overall study results). These should be interpreted only as suggested considerations and should not be viewed as scoring or a checklist. **Considering there are no controls in such designs, this should be evaluated carefully to be sure the report really contributes to the actual knowledge**.

**List of Considerations:**

***Possible sources of exposure assessment metrics:***

1) *Identification of the exposure*
2) *Dose evaluation*
3) *Toxicological values*
4) Clinical effects*
5) Biological effects*
6) *Treatments given (dose, timing, route)*

* *Some clinical and biological effects might be related to exposure*

***For each, overall considerations include:***

1) *What is the quality of the source of the metric being used?*
2) *Is the exposure measured in the study a surrogate for the exposure?*
3) *What was the temporal coverage (i.e., short or long-term exposure)?*
4) *Did the analysis account for prediction uncertainty?*
5) *How was missing data accounted for, and any data imputations incorporated?*
6) *Were sensitivity analyses performed?*

#### Were Outcome Assessment Methods Lacking Accuracy?

This item is similar to actual Navigation guidelines that require an assessment of the accuracy of the measured outcome.

#### Was Potential Confounding Inadequately Incorporated?

This is a very important issue for case reports/studies and case series. Case reports/studies and case series do not include controls and so, to be considered in a systematic review, these types of studies will need to be well-documented with respect to treatment or other contextual factors that may explain or influence the outcome. Prior to initiating the study screening, review team members should collectively generate a list of potential confounders that are based on expert opinion and knowledge gathered from the scientific literature:

*Tier I: Important confounders*

- Other associated treatment (i.e., intoxication, insufficient dose, history, or context)
- Medical history

*Tier II: Other potentially important confounders and effect modifiers:*

- Age, sex, country.

#### Were Incomplete Outcome Data Inadequately Addressed?

This item is similar to actual Navigation Guide instructions, though it may be very unlikely that outcome data would be incomplete in published case reports/studies and case series.

#### Does the Study Report Appear to Have Selective Outcome Reporting?

This item is similar to actual Navigation Guide instructions, though it may be very unlikely that there would be selective outcome reporting in published case reports/studies and case series.

#### Did the Study Receive Any Support From a Company, Study Author, or Other Entity Having a Financial Interest?

This item is similar to actual Navigation Guide instructions.

#### Did the Study Appear to Have Other Problems That Could Put It at a Risk of Bias?

This item is similar to actual Navigation Guide instructions.

### Data Synthesis Criteria and Summary Measures if Feasible

Meta-analyses will be conducted using a random-effects model if studies are sufficiently homogeneous in terms of design and comparator. For dichotomous outcomes, effects of associations will be determined by using risk ratios (RR) or odds ratios (OR) with 95% confidence intervals (CI). Continuous outcomes will be analyzed using weighted mean differences (with 95% CI) or standardized mean differences (with 95% CI) if different measurement scales are used. Skewed data and non-quantitative data will be presented descriptively. Where data are missing, a request will be made to the original authors of the study to obtain the relevant missing data. If these data cannot be obtained, an imputation method will be performed. The statistical heterogeneity of the studies using the Chi Squared test (significance level: 0.1) and I^2^ statistic (0–40%: might not be important; 30–60%: may represent moderate heterogeneity; 50–90%: may represent substantial heterogeneity; 75–100%: considerable heterogeneity). If there is heterogeneity, an attempt will be made to explain the source of this heterogeneity through a subgroup or sensitivity analysis.

Finally, the meta-analysis will be conducted in the latest version of the statistical software RevMan. The Mantel-Haenszel method will be used for the fixed effects model if tests of heterogeneity are not significant. If statistical heterogeneity is observed (*I*^2^ ≥ 50% or *p* < 0.1), the random effects model will be chosen. If quantitative synthesis is not feasible (e.g., if heterogeneity exists), a meta-analysis will not be performed and a narrative, qualitative summary of the study findings will be done.

Separate analyses will be conducted for the studies that contain control groups using expected mortality/morbidity, in order to include them in the quantitative synthesis of case reports/studies and case series.

If quantitative synthesis is not appropriate, a systematic narrative synthesis will be provided with information presented in the text and tables to summarize and explain the characteristics and findings of the included studies. The narrative synthesis will explore the relationship and findings both within and between the included studies.

### Possible Additional Analyses

If feasible, subgroup analyses will be used to explore possible sources of heterogeneity, if there is evidence for differences in effect estimates by country, study design, or patient characteristics (e.g., sex and age). In addition, sensitivity analysis will be performed to explore the source of heterogeneity as for example, published vs. unpublished data, full-text publications vs. abstracts, risk of bias (by omitting studies that are judged to be at high risk of bias).

### Overall Quality of Evidence Assessment

The quality of evidence will be assessed using an adapted version of the Evidence Quality Assessment Tool in the Navigation Guide. This tool is based on the GRADE approach (1). The assessment will be conducted by two teams, again blinded to each other, one that has the results of the case reports/studies and case series/control synthesis, the other without.

**Data synthesis will be conducted independently by the classical and case teams. Evidence ratings will start at “high” for randomized control studies, “moderate” for observational studies, and “low” for case reports/studies and case series**. It is important to be clear that sufficient levels of evidence cannot be achieved without study comparators. With regards to case reports/studies and case series, we classify these as starting at the lowest point of evidence and therefore we cannot consider evidence higher than low for these kinds of studies. Complete instructions for making quality of evidence judgments are presented in Supplementary Material.

### Synthesis of Strength of Evidence

The standard Navigation Guide methodology will be applied to rate the strength of recommendations. The classical and case teams, blinded to the results from each other during the process, will independently assess the strength of evidence. The evidence quality ratings will be translated into strength of evidence for each population based on a combination of four criteria: (a) Quality of body of evidence; (b) Direction of effect; (c) Confidence in effect; and (d) Other compelling attributes of the data that may influence certainty. The ratings for strength of evidence will be “sufficient evidence of harmfulness,” “limited of harmfulness,” “inadequate of harmfulness” and “evidence of lack of harmfulness.”

Once we complete the synthesis of case reports/studies and case series, findings of this separate evidence stream will only be considered if RCTs and observational studies are not available. They will not be used to upgrade or downgrade the strength of other evidence streams.

## Discussion

To the best of our knowledge, this protocol is one of the first to specifically address the incorporation of case reports/studies and case series in a systematic review (9). The protocol was adapted from the Navigation Guide with the intent of integrating the case reports/studies and case series in systematic review recommendations, while following traditional systematic review methodology to the greatest extent possible. To be included, these case report/studies and case series will need to be well-documented, scientifically rigorous, and follow ethical practices. In addition, we believe that some case reports/studies and case series might bring relevant knowledge that should be considered in systematic review recommendations when data from RCT's and observational studies are not available, especially when even a small number of studies report an important and possibly causal association in an epidemic or a side effect of a newly marketed medicine. Our methodology will be the first to effectively incorporate case reports/studies and case series in systematic reviews that synthesize evidence for clinicians, researchers, and drug developers. These types of studies will be incorporated mostly through paper selection and risk of bias assessments. In addition, we will conduct meta-analyses if the eligible studies provide sufficient data.

This protocol has limitations related primarily to the constraints of case reports/studies and case series. These are descriptive studies. In addition, a case series is subject to selection bias because the clinician or researcher selects the cases themselves and may represent outliers in clinical practice. Furthermore, this kind of study does not have a control group, so it is not possible to compare what happens to other people who do not have the disease or receive treatment. These sources of bias mean that reported results may not be generalizable to a larger patient population and therefore cannot generate information on incidences or prevalence rates and ratios (22, 23). However, it is important to note that promoting the need to synthesize these types of studies (case reports/studies and case series) in a formal systematic review, should not deter or delay immediate action from being taken when a few small studies report a plausible causal association between exposure and disease, such as, in the event of an epidemic or a side effect of a newly marketed medicine (23). In this study protocol, we will not consider animal studies that might give relevant toxicological information because we are focusing on study areas where a paucity of information exists. Finally, we must note that, case reports/studies and case series do not provide independent proof, and therefore, the findings of this separate evidence stream (case reports/studies and case series) will only be considered if evidence from RCTs and observational studies is not available. Case reports/studies and case series will not be used to upgrade or downgrade the strength of other evidence streams. In any case, it is very important to remember that these kinds of studies (case reports/studies and case series) are there to quickly alert agencies of the need to take immediate action to prevent further harm.

Despite these limitations, case reports/studies and case series are a first line of evidence because they are where new issues and ideas emerge (hypothesis-generating) and can contribute to a change in clinical practice (23–25). We therefore believe that data from case reports/studies and case series, when synthesized and presented with completeness and transparency, may provide important details that are relevant to systematic review recommendations.

## Author Contributions

AD and GS the protocol study was designed. JL, TW, and DM reviewed. MF, ALG, RV, NC, CB, GLR, MD, ML, and AN significant improvement was made. AN and AD wrote the manuscript. GS improved the language. All authors reviewed and commented on the final manuscript, read and approved the final manuscript to be published.

## Conflict of Interest

The authors declare that the research was conducted in the absence of any commercial or financial relationships that could be construed as a potential conflict of interest.

## Publisher's Note

All claims expressed in this article are solely those of the authors and do not necessarily represent those of their affiliated organizations, or those of the publisher, the editors and the reviewers. Any product that may be evaluated in this article, or claim that may be made by its manufacturer, is not guaranteed or endorsed by the publisher.
